# Rare case of multisystemic *Klebsiella pneumoniae* infection in a diabetic patient: a case report

**DOI:** 10.3389/fendo.2026.1691190

**Published:** 2026-02-12

**Authors:** Xiaochang Bin, Fen Tang, Li Zou, Jian Lv, Yanjie Luo, Zhenqiang Tang

**Affiliations:** 1 Specialized Medical Care Unit, Nanxishan Hospital of Guangxi Zhuang Autonomous Region (The Second People’s Hospital of Guangxi Zhuang Autonomous Region), Guilin, Guangxi Zhuang Autonomous Region, China; 2 Clinical Pharmacy, Nanxishan Hospital of Guangxi Zhuang Autonomous Region (The Second People’s Hospital of Guangxi Zhuang Autonomous Region), Guilin, Guangxi Zhuang Autonomous Region, China

**Keywords:** case report, diabetes mellitus, follow-up, *Klebsiella pneumoniae*, multisystemic infection

## Abstract

**Background:**

Multisystemic infections caused by *Klebsiella pneumoniae* (Kp) are rare but can be life-threatening, particularly in patients with diabetes mellitus (DM). We report a case of a 65-year-old diabetic female patient who developed concurrent ocular, intracranial, hepatic, and pulmonary abscesses caused by *K. pneumoniae*.

**Case presentation:**

The patient presented with a subacute onset of cough, fever, and ocular symptoms. The diagnosis was confirmed through integrated clinical assessment, laboratory analysis, and multimodal imaging. The patient’s condition was momentarily improved following enucleation of the infected eye and initial empirical therapy with meropenem and vancomycin, followed by targeted antimicrobial treatment based on the microbiological results. Regular follow-up with serial cranial magnetic resonance imaging (MRI) scans was performed to monitor disease progression and response to treatment.

**Conclusion:**

This case highlights the critical necessity for early diagnosis, aggressive surgical intervention, and prolonged antimicrobial therapy in diabetic patients with severe Kp dissemination. Comprehensive clinical and radiological follow-up is paramount to ensure therapeutic efficacy and recovery.

## Introduction

*Klebsiella pneumoniae* (Kp), a clinically significant Gram-negative bacteria, causes severe infections with heightened risk in immunocompromised individuals—including those with diabetes, malignancies, and chronic kidney disease or recipients of immunosuppressive therapies or organ transplantation (1). K*. pneumoniae*, as a global health concern, is marked by its association with primary liver abscesses and invasive metastatic complications (1, 2). Epidemiological evidence indicates that hematogenous dissemination occurs in liver abscess cases, leading to life-threatening sequelae such as cerebral abscesses, necrotizing pneumonia, endogenous endophthalmitis, or necrotizing fasciitis, substantially elevating morbidity and mortality (3). Consequently, early diagnosis and comprehensive management are critical for optimizing outcomes (4). This case report details a disseminated *K. pneumoniae* infection across multiple organ systems to demonstrate the clinical utility of multidisciplinary team (MDT) collaboration in complex infection management, establish a standardized follow-up protocol for treatment monitoring and relapse prevention, and delineate a transferable diagnostic–therapeutic framework for *K. pneumoniae* infections.

## Case presentation

The patient was a 65-year-old Chinese woman with a history of poorly controlled type 2 diabetes mellitus. The patient had a 12-year history of type 2 diabetes, managed with oral metformin (500 mg twice daily) without regular blood glucose monitoring. On admission, her glycated hemoglobin (HbA1c) was 10.8% (reference range: 4.0%–6.5%), and she had diabetic peripheral neuropathy (decreased sensation in both lower extremities) without diabetic nephropathy or retinopathy unrelated to this infection. The patient was admitted to the hospital on January 29, 2025 due to a subacute onset of cough, fever, and ocular symptoms. She had a 14-day history of productive cough with yellowish purulent sputum and 5 days of intermittent fever (peak temperature, 39.6 °C). Ocular symptoms, which appeared on January 24, 2025, included progressive visual loss in the left eye (from 0.3 at onset to hand motion visual acuity on admission), severe ocular pain (visual analog scale [VAS] score 7/10), eyelid swelling, conjunctival hyperemia and edema, purulent discharge, and exacerbated pain with eye movement. Ophthalmic examination revealed left eye endophthalmitis, with corneal edema and opacity, 2 mm hypopyon in the anterior chamber, and significant vitreous opacity. Initial assessment noted a tachycardia of 112 beats/min, tachypnea of 22/min, despite normothermia (37.0 °C), and blood pressure of 127/90 mmHg. Physical examination revealed an alert, cooperative patient without jaundice, respiratory distress, or peripheral edema. Cardiopulmonary auscultation demonstrated regular rhythm without murmur. The abdomen was soft, with no tenderness, rebound, or guarding. The neurological examination was nonfocal.

### Laboratory and molecular biology tests

Laboratory investigations revealed systemic inflammation: white blood cell (WBC) count at 16.22 × 10^9^/L (reference range: 3.50–9.50 × 10^9^/L), C-reactive protein (CRP) at 151.51 mg/L (reference range: 0–10 mg/L), procalcitonin (PCT) at 7.73 ng/mL (reference range: 0–0.15 ng/mL), and erythrocyte sedimentation rate (ESR) 86 mm/h (reference range: 0–20 mm/h).

### Pathogen isolation and identification

Blood cultures, ocular swabs, and hepatic abscess drainage fluid (pus) were collected for microbiological testing. Specimens were inoculated on blood agar and MacConkey agar plates and cultured at 35 °C for 24–48 h. Gram-negative bacteria were isolated from all specimens: *Klebsiella pneumoniae* was identified from blood, ocular swabs, and hepatic abscess drainage fluid (VITEK 2 Compact system, identification rate = 99.9%). Repeat testing confirmed *Klebsiella pneumoniae* as the sole pathogen.

### Hypermucoviscous and hypervirulent *K. pneumoniae* verification

Phenotypic test: The *K. pneumoniae* isolate showed a negative string test result (no mucous thread ≥5 mm formed when stretched with an inoculating loop), which is inconsistent with the typical hypermucoviscous phenotype of hypervirulent *Klebsiella pneumoniae* (hvKp) (5).
Genotypic test: PCR detection of hvKp-specific virulence genes (rmpA, aerobactin) was not performed in clinical routine testing.
Antimicrobial susceptibility testing: Kirby–Bauer method was used for antimicrobial susceptibility testing. *K. pneumoniae* was sensitive to multiple antibiotics, including amoxicillin/clavulanate (MIC ≤2.0 μg/mL), piperacillin/tazobactam (MIC ≤4.0 μg/mL), ceftazidime (MIC 0.25 μg/mL), imipenem (MIC ≤0.25 μg/mL), and meropenem (MIC ≤0.12 μg/mL). Extended-spectrum β-lactamase (ESBL) production was negative.

### Imaging studies

**Figure 1:**
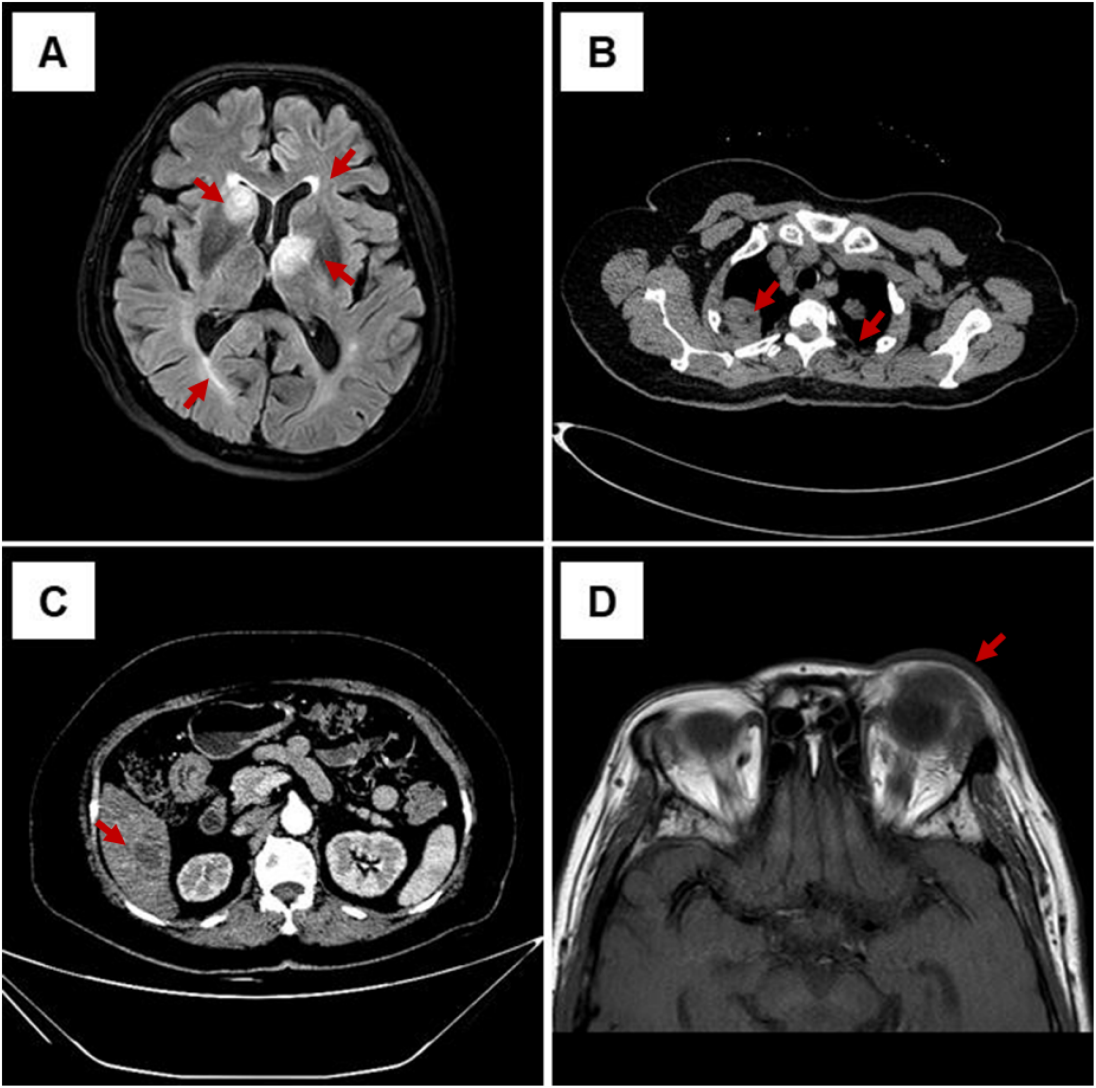
Initial multimodal imaging demonstrating multisystemic abscesses at presentation. **(A)** Cranial MRI FLAIR sequence, axial plane, slice thickness = 5 mm: four intracranial abscesses (marked with red arrows) in the left frontal horn, right occipital horn of the lateral ventricle, right caudate nucleus, and left thalamus, with surrounding edema. **(B)** Pulmonary CT, axial plane, mediastinal window (window width = 300 HU, window level = 40 HU), slice thickness = 1 mm: pulmonary abscesses (marked with red arrows) in both lungs, with necrosis and cavitation. **(C)** Abdominal CT, axial plane, liver window (window width = 200 HU, window level = 50 HU), slice thickness = 1 mm: a large hepatic abscess (marked with red arrows) in the right lobe of the liver, with liquefaction and necrosis. **(D)** Ocular MRI, axial T1 post-contrast, slice thickness = 3 mm: increased vitreous signal and thickened ocular wall in the left eye, consistent with endophthalmitis.

Serial computed tomography (CT) and magnetic resonance imaging (MRI) scans were performed on admission. The detailed imaging characteristics were as follows: cranial MRI FLAIR sequence (**Figure 1A**), axial plane, slice thickness = 5 mm: Four intracranial abscesses were identified in the left frontal horn of the lateral ventricle, right occipital horn of the lateral ventricle, right caudate nucleus, and left thalamus, with surrounding cerebral edema (marked with red arrows). Pulmonary CT (**Figure 1B**): axial plane, mediastinal window (window width = 300 HU, window level = 40 HU), slice thickness = 1 mm: Pulmonary abscesses were detected in both lungs, with necrosis and cavitation (marked with red arrows). Abdominal CT (**Figure 1C**): Axial plane, liver window (window width = 200 HU, window level = 50 HU), slice thickness = 1 mm: A large hepatic abscess was found in the right lobe of the liver, with liquefactive necrosis (marked with red arrows). Ocular MRI T1-weighted post-contrast sequence (**Figure 1D**): Axial plane, slice thickness = 3 mm: Increased signal intensity in the vitreous body of the left eye and thickening and enhancement of the ocular wall, consistent with endophthalmitis. All MRI images were independently reviewed by two associate chief radiologists, with consistent diagnoses. Image quality met clinical assessment requirements and supported the diagnosis of multisystemic abscesses.

### Process of treatment

On hospital day 7 (February 5, 2025), ultrasound-guided percutaneous hepatic abscess drainage was performed using the Seldinger technique. An 8Fr drainage tube was inserted, and approximately 150 mL of yellowish green purulent fluid was drained. Culture of the drainage fluid confirmed *K. pneumoniae* (consistent with blood culture in antimicrobial susceptibility pattern), with no *E. coli* detected. Continuous drainage was maintained until hospital day 14, when the drainage fluid became clear. Repeat abdominal CT showed that the abscess cavity had shrunk to less than 2 cm, and the drainage tube was removed.

On hospital day 3, an ophthalmology consultation was requested (assessed by an associate chief ophthalmologist). Following diagnosis, intensive conservative management was initiated, including systemic broad-spectrum intravenous antibiotics and supportive ophthalmic care. Despite these measures, the patient experienced persistent severe ocular pain, progressive visual deterioration, and radiological evidence of uncontrolled intraocular infection, indicating poor response to conservative treatment. Based on ocular symptoms, signs, and MRI findings, the patient was diagnosed with severe infectious endophthalmitis in the left eye. Given the poor visual prognosis (hand motion visual acuity, hypopyon, and severe vitreous opacity) and the eye being a potential source of systemic infection with ongoing dissemination risk, a multidisciplinary team (MDT) discussion involving infectious disease specialists, surgeons, ophthalmologists, and endocrinologists was conducted. It was determined that conservative treatment could not effectively control the ocular infection or systemic dissemination, so enucleation of the infected left eye was performed on hospital day 10 (February 8, 2025). Pathological examination of the enucleated eye specimen showed diffuse inflammatory infiltration of intraocular tissues with *K. pneumoniae* colonies identified.

Empirical intravenous antimicrobial therapy with meropenem (2 g every 8 h) combined with vancomycin (1 g every 12 h) was initiated on hospital day 1 (January 30, 2025), pending microbiological identification. The empirical regimen was chosen based on the following considerations:

1. High suspicion of Gram-negative bacterial infection (e.g., *K. pneumoniae*, *E. coli*) in a diabetic patient with multisystemic abscesses; meropenem, a carbapenem antibiotic, has potent activity against Enterobacteriaceae and can cross the blood–brain and blood–ocular barriers, making it suitable for intracranial and ocular infections.
2. Increased risk of concurrent Gram-positive bacterial infection (e.g., *Staphylococcus*, *Streptococcus*) in immunocompromised diabetic patients, particularly in endophthalmitis where Gram-positive pathogens are common; vancomycin was included empirically to provide coverage against potential Gram-positive pathogens, which are common causes of severe endophthalmitis, until microbiological results excluded Gram-positive infection.
3. Subsequent microbiological and antimicrobial susceptibility results confirmed the efficacy of the regimen. Once cultures consistently identified *Klebsiella pneumoniae* as the sole pathogen, antimicrobial therapy was de-escalated accordingly, with continuation of targeted treatment. Glycemic control was optimized with insulin therapy throughout the treatment course.

### Clinical outcomes and follow-up

**Figure 2:**
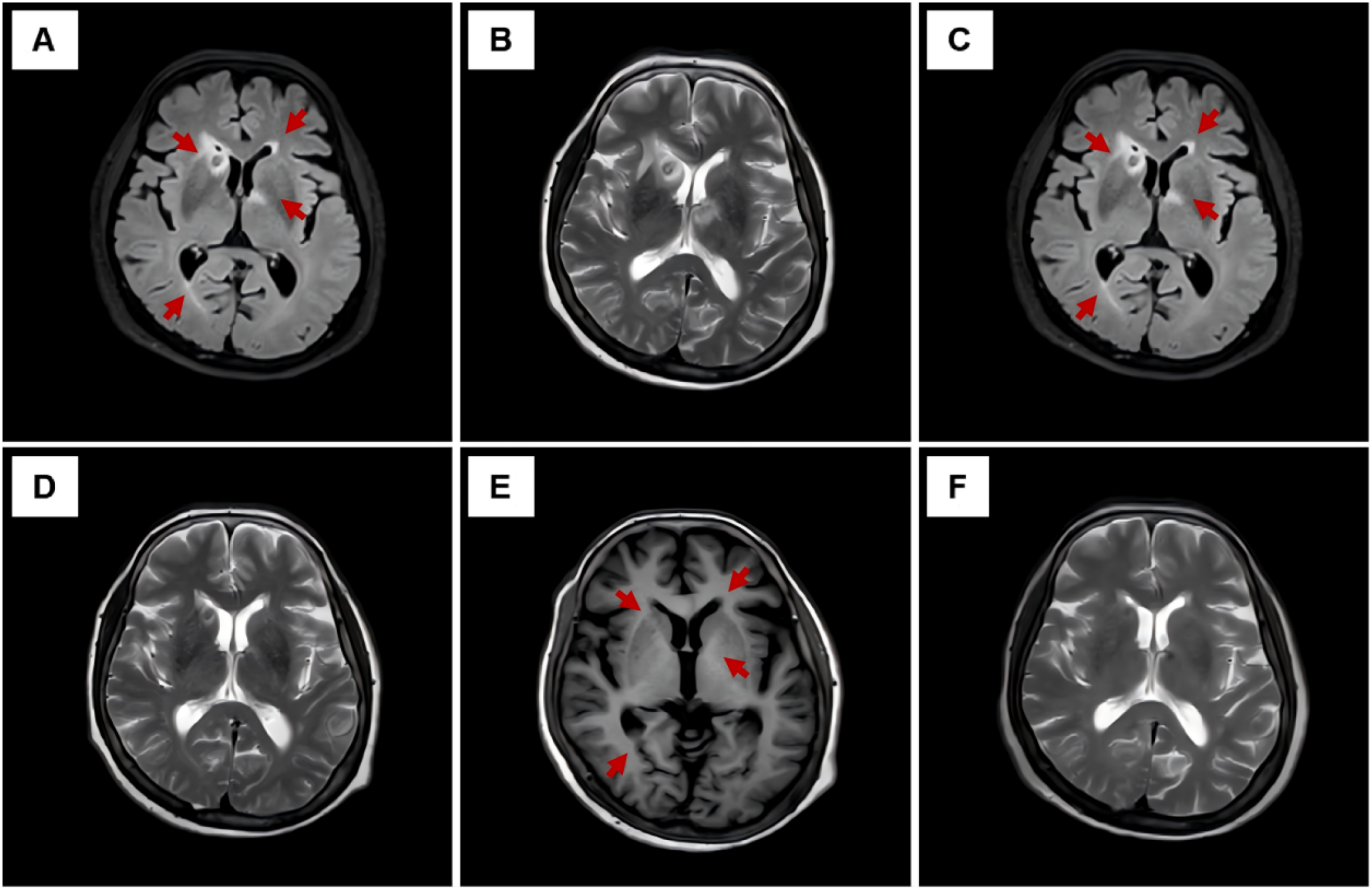
Serial cranial MRI findings during treatment and follow-up. **(A)** Cranial MRI FLAIR unenhanced sequence (axial plane, slice thickness = 5 mm) at week 4 showing mild abscess enlargement and progressive perilesional edema (red arrows) consistent with active inflammation. **(B)** Cranial MRI T2-weighted unenhanced sequence (axial plane, slice thickness = 5 mm) at week 6 demonstrating persistent abscess boundaries consistent with peak perilesional edema observed clinically. **(C)** Cranial MRI FLAIR unenhanced sequence (axial plane, slice thickness = 5 mm) at week 8 showing the initial regression of perilesional edema and a significant decrease in abscess size (red arrows), indicating the onset of abscess resolution. **(D)** Cranial MRI T2-weighted unenhanced sequence (axial plane, slice thickness = 5 mm) at week 10, revealing further reduction in abscess size consistent with advanced resolution. **(E)** Cranial MRI FLAIR unenhanced sequence (axial plane, slice thickness = 5 mm) at week 12 demonstrating near-complete absorption of intracranial abscesses, with only minimal residual fibrosis and no significant edema (red arrows). **(F)** Cranial MRI T2-weighted unenhanced sequence (axial plane, slice thickness = 5 mm) at week 20 confirming the complete resolution of all intracranial abscesses without evidence of recurrence. **(A, C, E)** are FLAIR unenhanced sequences. **(B, D, F)** are T2-weighted unenhanced sequences.

**Figure 3:**
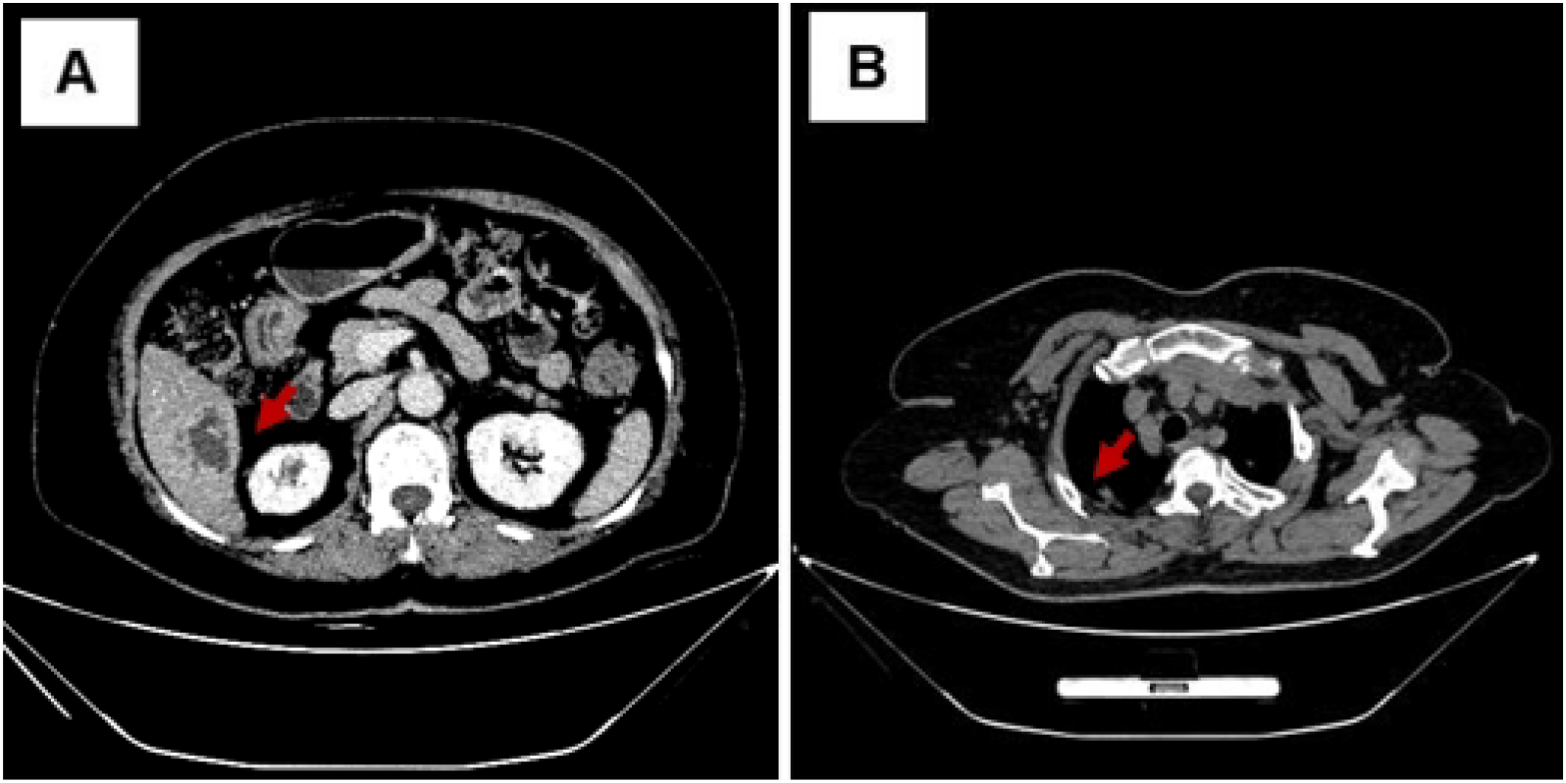
Follow-up abdominal and pulmonary CT imaging of the patient at week 10. **(A)** Abdominal CT (liver window, window width = 200 HU, window level = 50 HU, axial plane, slice thickness = 1 mm): The hepatic abscess was completely absorbed, with no residual areas of necrosis or fluid collection. **(B)** Pulmonary CT (mediastinal window, window width = 300 HU, window level = 40 HU, axial plane, slice thickness = 1 mm): The pulmonary abscesses significantly decreased in size, with absorption of necrotic areas and improvement in lung parenchyma.

Regular follow-up with serial cranial MRI examinations at predefined intervals was conducted to monitor the resolution of intracranial abscesses. Cranial MRI FLAIR sequence (axial plane, slice thickness 5 = mm) at week 4 demonstrated progressive perilesional edema and mild abscess enlargement (**Figure 2A**). At week 6, T2-weighted unenhanced imaging (**Figure 2B**) confirmed persistent abscess boundaries. From week 8 onward, FLAIR imaging (**Figure 2C**) revealed an initial regression of perilesional edema and a significant decrease in abscess size. By week 10, T2-weighted imaging (**Figure 2D**) showed a further reduction in abscess volume, and FLAIR imaging at week 12 (**Figure 2E**) demonstrated near-complete absorption of intracranial abscesses, leaving only minimal residual fibrosis without significant edema. Clinical improvement, including defervescence and normalization of inflammatory markers (CRP), paralleled radiological resolution. Abdominal CT (liver window, window width = 200 HU, window level = 50 HU, axial plane, slice thickness = 1 mm) and pulmonary CT (mediastinal window, window width = 300 HU, window level = 40 HU, axial plane, slice thickness = 1 mm) at week 10 demonstrated complete absorption of the hepatic abscess (**Figure 3A**) and a marked reduction of pulmonary cavities (**Figure 3B**). Final cranial MRI T2-weighted sequence (axial plane, slice thickness = 5 mm) at week 20 confirmed the complete resolution of all intracranial abscesses without recurrence (**Figure 2F**) (**Table 1**).

**Table 1:** Timeline of clinical events.

Date	Clinical events
January 15, 2025	Onset of cough with yellow purulent sputum (initial symptom)
January 20, 2025	Worsening cough accompanied by fever (38.5 °C) Self-administered oral cephalosporins (unspecified) without clinical improvement
January 24, 2025	Development of decreased visual acuity in the left eye, ocular pain, eyelid erythema, and swelling; fever aggravated (peak temperature 39.6°C) with headache
January 29, 2025	Hospital admission due to progressive symptoms; comprehensive diagnostic evaluation performed
January 30, 2025	Blood cultures and ocular swab Gram staining suggested Gram-negative bacteria; empirical antibiotic therapy with meropenem and vancomycin initiated
February 2, 2025	Definitive microbiological results identified *Klebsiella pneumoniae* (*Escherichia coli* not detected).
February 5, 2025	Percutaneous drainage of liver abscess performed
February 8, 2025	Left eye enucleation performed due to uncontrolled infection
February–April 2025	Continued targeted antimicrobial therapy; serial brain MRI examinations performed at predefined intervals for follow-up
April 8, 2025	Completion of antimicrobial therapy (total duration: 10 weeks); follow-up imaging showed marked absorption of lesions
June 17, 2025	Final follow-up (approximately 20 weeks after initiation of antimicrobial therapy); imaging demonstrated complete resolution of abscesses

Based on the clinical timeline, the lungs were suspected as the possible initial site of infection given the early presentation with respiratory symptoms; yet, alternative scenarios, such as an initially asymptomatic hepatic abscess or early bacteremia preceding overt organ involvement, cannot be excluded. Subsequently, hematogenous dissemination occurred to the liver, eyes, and intracranial cavity, consistent with the typical pathway of disseminated *K. pneumoniae* infection. The sequential development of multi-organ abscesses indicates progressive dissemination, highlighting the importance of early intervention.

## Discussion

### Case uniqueness

This case exhibits distinct characteristics compared to previous reports, namely:

Extensive multisystem involvement: Concurrent abscess formation involving four organ systems—the eye, brain, liver, and lung—is exceedingly rare in disseminated *K. pneumoniae* infections. Classic hypervirulent *K. pneumoniae* (hvKp) infections typically present with a primary liver abscess followed by metastatic spread to distant sites, most commonly the eye or the central nervous system (6, 7). In contrast, the present case initially manifested with pulmonary symptoms and subsequently progressed to four-organ involvement, suggesting an atypical dissemination pattern with a broader scope of systemic involvement.
Single-pathogen-driven extensive dissemination: While single-pathogen *Klebsiella pneumoniae* infection is common in disseminated cases (8, 9), its progression to four-organ abscess formation (eye, brain, liver, and lung) is rarely documented, which distinguishes this case from typical disseminated infections that usually involve one to two organ systems (10, 11).
Absence of a clear portal of entry: Most reported disseminated *Klebsiella pneumoniae* infections have an identifiable primary source, such as the gastrointestinal or biliary tract (9). In this case, extensive investigations (including serial abdominal CT, laboratory tests, and microbiological cultures) failed to identify a definite primary focus. The findings suggest hematogenous dissemination from an occult primary focus (12), which is uncommon in non-hvKp disseminated infections and further underscores the rarity of this presentation.

Compared with the case reported by Siu et al. (13), in which a liver abscess served as the primary source of multi-organ infection, the present case lacked evidence of a primary hepatic focus and was caused by a single pathogen (*Klebsiella pneumoniae*). In comparison with Doshi et al. (8), whose case involved three organ systems, this case exhibited more extensive organ involvement (four systems), necessitating a more complex and aggressive treatment strategy.

Compared with previously reported cases, this case provides a more comprehensive evidence chain, including concordant isolation of *Klebsiella pneumoniae* from three independent sites (blood, ocular tissue, and hepatic abscess), systematic multimodal imaging documenting multisystem involvement, clearly defined source control interventions, and longitudinal radiological follow-up demonstrating complete resolution. These elements strengthen causal inference and therapeutic attribution beyond isolated case descriptions.

### Pathophysiology mechanisms and clinical significance

This case demonstrates multisystem abscesses due to *K. pneumoniae* in a patient with poorly controlled diabetes. Diabetes impairs immune function through hyperglycemia-induced defects in macrophage activity, T-cell response, and cytokine production (14, 15). Hyperglycemia also promotes bacterial growth and biofilm formation (16), while microvascular dysfunction compromises local immune surveillance (17). Notably, the *K. pneumoniae* isolate was string-test negative, indicating a non-hypermucoviscous phenotype. Nevertheless, the clinical course—marked by rapid progression, simultaneous multi-organ involvement, and severe metastatic sites (endophthalmitis, intracranial abscess)—aligns with hvKp-like disease. Recent evidence indicates that string test negativity does not exclude hvKp, as the test has limited sensitivity and does not capture the full virulence spectrum (18). Although virulence gene testing was not performed, the aggressive phenotype supports a mechanistic explanation for the severe dissemination. Endophthalmitis and CNS involvement are particularly morbid manifestations of hvKp (19), underscoring the need for early recognition. Therefore, the diagnosis of hypervirulence in this case was based on clinical phenotype rather than molecular confirmation.

### Predisposing factors and portal of entry

The key risk factors included (1) poor long-term glycemic control (HbA1c, 10.8%), (2) diabetic peripheral neuropathy, potentially delaying symptom recognition, and (3) inappropriate pre-admission antibiotic use. With no history of trauma or surgery, an endogenous route—likely from gut or respiratory colonization—was presumed. The absence of an identified primary focus reinforces the unusual nature of this presentation.

Early diagnosis is challenging due to non-specific symptoms. Blood cultures and comprehensive imaging are crucial (20, 21). Here concurrent ocular, neurological, and respiratory symptoms prompted a multidisciplinary approach, enabling timely identification.

Management required aggressive source control combined with prolonged antibiotics. Enucleation removed an uncontrolled focus, and ultrasound-guided hepatic abscess drainage provided both therapeutic and diagnostic value.

### Diagnostic challenges and treatment strategy

Empirical antimicrobial treatment with meropenem and vancomycin was initiated and later refined based on microbiological results. Meropenem was selected for its broad-spectrum activity against Gram-negative pathogens and its excellent penetration of the blood–brain and blood–ocular barriers (22), while vancomycin was included to cover potential Gram-positive organisms, particularly in the context of severe endophthalmitis (23). A prolonged 10-week course of antimicrobial therapy was essential for the complete eradication of infection and prevention of relapse, consistent with treatment principles for severe invasive *K. pneumoniae* infection with suspected hypervirulent features (24).

Strict glycemic control with insulin therapy during hospitalization and follow-up was another critical component in achieving successful management. Hyperglycemia is associated with poor outcomes in patients with *Klebsiella pneumoniae* infections, including liver abscesses, as it impairs immune function and affects the efficacy of antimicrobial therapy. Several studies suggest that hyperglycemia in diabetic patients with *K. pneumoniae* infections may lead to worse outcomes due to immune dysfunction and impaired bacterial clearance (25).

Although genotypic virulence testing was not routinely performed, the clinical phenotype warranted management according to principles for severe invasive *K. pneumoniae* infection with suspected hypervirulent features, including aggressive source control and prolonged antimicrobial therapy.

### Importance of follow-up

Comprehensive clinical and radiological follow-up is crucial in the management of severe disseminated infections. In this case, biweekly cranial MRI examinations allowed a dynamic assessment of intracranial abscess resolution and directly informed decisions regarding the duration of antimicrobial therapy. Serial monitoring of inflammatory markers, including C-reactive protein and procalcitonin, provided complementary indicators of treatment response and correlated well with radiological improvement.

At 20 weeks of follow-up, complete radiological resolution of all abscesses was confirmed, with no evidence of recurrence, validating the effectiveness of the multidisciplinary and individualized treatment strategy.

## Conclusion

This case report highlights the successful management of a rare and severe multisystemic *Klebsiella pneumoniae* infection in a patient with poorly controlled diabetes mellitus. Early diagnosis, aggressive source control, prolonged targeted antimicrobial therapy, strict glycemic control, and structured clinical and radiological follow-up were critical to achieving complete recovery. Multidisciplinary team involvement, including infectious disease specialists, surgeons, radiologists, ophthalmologists, and endocrinologists, was essential for optimizing outcomes. This case underscores the importance of vigilance in diabetic patients presenting with systemic infection and demonstrates the value of individualized follow-up strategies in guiding treatment duration and preventing relapse.

## Data Availability

The original contributions presented in the study are included in the article/supplementary material. Further inquiries can be directed to the corresponding author.
